# Diagnostic accuracy of the neurological upper limb examination II: Relation to symptoms of patterns of findings

**DOI:** 10.1186/1471-2377-6-10

**Published:** 2006-02-27

**Authors:** Jørgen R Jepsen, Lise H Laursen, Carl-Göran Hagert, Svend Kreiner, Anders I Larsen

**Affiliations:** 1 Department of Occupational Medicine, Sydvestjysk Sygehus, Østergade 81–83, DK-6700 Esbjerg, Denmark; 2 Department of Orthopaedic Surgery, University Hospital, S-22185 Lund, Sweden; 3 Department of Biostatistics, University of Copenhagen, DK-2200 Copenhagen, Denmark; 4 Occupational Health Services, Novozymes, DK-2880 Bagsværd, Denmark

## Abstract

**Background:**

In a sample of patients in clinical occupational medicine we have demonstrated that an upper limb neurological examination can reliably identify patterns of findings suggesting upper limb focal neuropathies. This further study aimed at approaching the diagnostic accuracy of the examination.

**Methods:**

82 limbs were semi-quantitatively assessed by two blinded examiners (strength in 14 individual muscles, sensibility in 7 homonymous territories, and mechanosensitivity at 10 locations along nerves). Based on the topography of nerves and their muscular and sensory innervation we defined 10 neurological patterns each suggesting a localized nerve affliction. Information on complaints (pain, weakness and/or numbness/tingling) collected by others served as a reference for comparison. The relation between the presence of pattern(s) and complaints was assessed by κ-statistics. Sensitivity, specificity, and positive/negative predictive values were calculated, and pre-test odds were compared to post-test probability.

**Results:**

The two examiners identified pattern(s) suggesting focal neuropathy in 34/36 out of 38 symptomatic limbs, respectively (κ = 0.70/0.75), with agreement in 28 limbs. Out of 44 non-symptomatic limbs the examiners agreed on absence of any pattern in 38 limbs. With concordance between the examiners with regard to the presence or absence of any pattern, the sensitivity, specificity, positive and negative predictive values were 0.73, 0.86, 0.93 and 0.90, respectively. While the pre-test odds for a limb to be symptomatic amounted to 0.46 the post-test probability was 0.81. For each examiner the post-test probability was 0.87 and 0.88, respectively.

**Conclusion:**

The improved diagnostic confidence is an indication of one aspect of construct validity of the physical examination. For determination of clinical feasibility of the examination further studies are required, most importantly 1) studies of validity by means of comparison with additional references and 2) studies of the potential benefit that can be attained from its use.

## Background

Potentially work-related upper limb disorders remain diagnostic challenges when the standard physical examination cannot identify well-described clinical conditions [1]. In many patients the character of pain and the accompanying subjective motor and sensory disturbances suggest a peripheral nerve-involvement.

We have formerly documented the inter-rater reproducibility of manual testing of the strength in 14 individual upper limb muscles and the relation of identified weaknesses to symptoms [2]. The additional inclusion in a neurological examination of an evaluation of sensory disturbances and mechanosensitivity of nerve trunks has permitted the reliable identification of patterns relating to the innervation and course of the peripheral nerves.

Clinical significance, however, demands the additional demonstration of validity in terms of relating physical abnormalities to other aspects of disease, health status or condition. One example of the latter is the presence of diffuse upper limb pain, weakness, and/or numbness/tingling which are characteristic features of peripheral nerve afflictions. This study has estimated the relation between these complaints and the occurrence in defined patterns of the implicated physical parameters.

## Methods

### Participants

Consecutive patients with any disorder (upper limb, low back, lung, etc.) attending the Department of Occupational Medicine, Sydvestjysk Sygehus Esbjerg were considered for enrolment in the study. The department is a secondary referral centre for assessment of the work-relatedness of any disorder and of consequences regarding work capacity.

**Figure 1:**
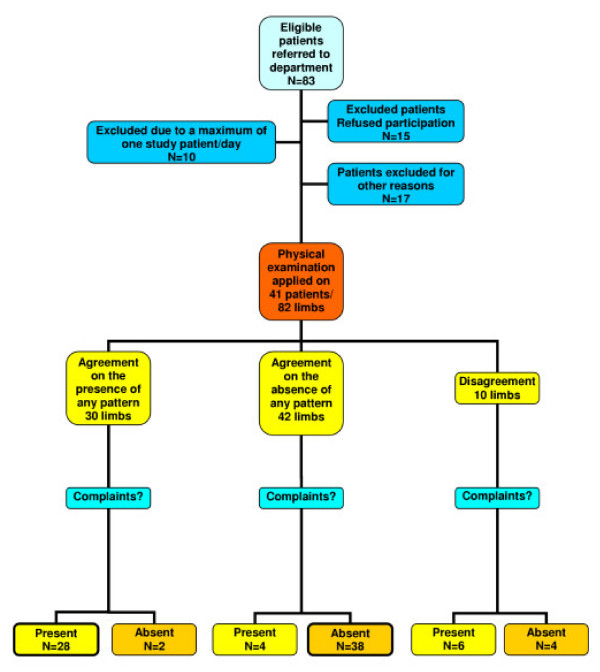
Flow diagram illustrating the patient-sample.

In order to secure instructions and blinding, patients were excluded when known from earlier contacts to the department, when speaking a foreign language or when presenting visible indication to suggest an assignment to the symptomatic or the non-symptomatic group such as scars from prior upper limb surgery or an appearance suggesting recognizable disease, e.g. an antalgic position. The study sample was limited to the first eligible patient each day during the study and constituted 41 patients/82 limbs (Figure 1). Based on presuppositions with regard to the distribution of deviations from normal of the physical findings, this sample size was determined as adequate for statistical calculations of sufficient power. Data were collected prospectively.

The study complied with the Helsinki declaration. It was approved by the local Ethics Committee and signed informed consent was obtained from all participants.

### Test methods

All 41 patients underwent two sets of clinical examinations:

• One out of two *primary *examiners performed an interview followed by a "conventional" physical screening examination [3] excluding the items studied by the secondary examiners.

• Two *secondary *examiners performed identical physical examinations targeting the upper limb peripheral nerves.

#### Primary examination

Two *primary *examiners, both board-certified specialists in occupational medicine, each interviewed half of the patients about upper limb symptoms. Each limb was classified with respect to the presence or absence of pain, weakness, and/or numbness/tingling.

Subsequently, they performed a physical screening examination including the range of motion of individual joints together with the recording of pain responses and the palpation of tendons, insertions and muscles. The assessment of individual muscle strength, sensibility at various innervation territories and mechanosensitivity of nerves was excluded. The history, physical examination and extracts of physical criteria permitted the identification of the following clinical conditions: Tension neck syndrome, cervical syndrome, supra- and infraspinatus tendonitis, bicipital tendonitis, frozen shoulder, acromioclavicular arthritis, epicondylitis, and wrist and forearm tenosynovitis/peritendonitis [3]. The primary examiner stated in each limb the presence or absence of these disorders.

#### Blinded physical examination and diagnostic interpretation

Subsequently, two *secondary *examiners (authors: JRJ and LHL) situated alone at a distance performed independent and identical neurological examinations in immediate succession one after the other. Both were blinded to any patient characteristics including the results of the initial examination. No communication was allowed during this part of the examination except instructions to patients and their responses to the applied tests. The examinations comprised the following parameters:

#### • Muscle strength

The voluntary isometric strength was manually assessed in 14 individual upper limb muscles considered to be sufficiently accessible and representative of the upper limb nerves: Posterior deltoid, pectorals, latissimus dorsi, biceps, triceps, infraspinatus, short radial extensor of wrist, radial flexor of wrist, long flexor of thumb, long extensor of thumb, short abductor of thumb, ulnar extensor of wrist, deep flexor of 5^th ^finger, and abductor of 5^th ^finger. The examination was performed systematically with comparison right and left from proximal to distal as proposed by one of the authors (C-GH). The limb was positioned and stabilized to achieve an approximately isolated action of each muscle studied [2].

#### • Sensibility

The sensibility to moving touch [4] and pinprick was examined in 7 homonymously innervated upper limb territories: Axillary, medial cutaneous of arm and of forearm, musculocutaneous, radial, median, and ulnar. The perception of vibration was assessed by a tuning fork 256 Hz [5] at the volar tips of the 2^nd ^and 5^th ^finger. Deviation of sensibility was classified as "marked" when an allodynic reaction was recorded, or when touch, pain or vibration could either not be perceived at all or was reduced sufficiently to be clearly apparent to the examiner from the patient's reaction. Deviation of sensibility was classified as "mild/any" with any other divergence from normal (hypo- or hypersensibility). For the latter assessment, findings were compared with sensibility in other territories assessed as normal.

#### • Mechanosensitivity of nerve trunks

Nerves were examined for mechanical allodynia [6-9] with a manual pressure of 3 kp at 10 locations from proximal to distal along the nerves: The brachial plexus at the scalene triangle and at the passage behind the pectoralis minor muscle, the suprascapular nerve at the suprascapular notch, the axillary nerve in the quadrilateral space, the musculocutaneous nerve at the passage through the coracobrachial muscle, the radial/posterior interosseous nerve at the triceps and brachioradialis arcades, radiohumeral joint and supinator tunnel, the median nerve at elbow level and at the carpal tunnel, and the ulnar nerve at elbow level. "Marked" mechanical allodynia was registered with avoidance reaction/jump sign, "medium" allodynia when the patient expressed the pressure as seriously uncomfortable, and "mild/any" allodynia with the presence of any other soreness regarded as exceeding normal. For the latter assessment, the level of mechanical allodynia was compared to reactions regarded as normal to pressure elsewhere along nerves.

All physical findings were semi-quantitatively rated (Table 1). Based on the course and innervation patterns of the peripheral nerves, we defined 10 patterns each of which were assigned to a specific location (Table 2). Each limb was characterized in terms of presence or absence of any pattern (Table 3 and 4).

#### Reference standard

Complaints of pain, weakness and/or numbness/tingling were chosen as references for comparison in this study.

### Statistics

The relationship between the presence of any of the defined patterns and complaints (pain, weakness and/or numbness/tingling) was assessed by Cohen's κ statistics, a measure for testing whether agreement between raters of categorical data exceeds chance levels: κ = (p_o _- p_e_)/(1 - p_e_) where p_o _is the proportion of observed agreement and p_e _is the proportion of agreement expected by chance. The κ coefficient has a maximum of 1.0 and is interpreted as κ: < 0.2 = poor, 0.21–0.40 = fair, 0.41–0.60 = moderate, 0.61–0.80 = good, 0.81–1.00 = very good [10].

In limbs with agreement between both examiners, we determined the diagnostic sensitivity, specificity, and the positive and the negative predictive values of the combined tests in relation to complaints. In addition, we calculated the pre-test odds = prevalence of complaints/(1 - prevalence of complaints), the likelihood ratio for a positive test = sensitivity/(1- specificity), and the post-test odds = pre-test odds x likelihood ratio [10]. The post-test probability (the diagnostic confidence of the blinded physical examination in relation to complaints expressed as the post-test odds/(post-test odds + 1) was compared to the pre-test odds. Similar calculations were performed for each secondary examiner. A good diagnostic test is reflected by a high difference between the pre-test odds and the post-test probability.

### Role of the funding source

The funding sources have had no role in the study design, the collection, analysis and interpretation of data, and the decision to submit for publication.

## Results

### Participants

41 patients recruited between January 5^th ^and May 20^th ^1998 satisfied the inclusion criteria and participated in the index tests (Figure 1). 22 were males of median age 44 (range 29–61) years, and 19 females of median age 39 (range 25–52) years. Prior diagnostic difficulties, no responses to prior treatment or a recurrence of symptoms on resuming work were characteristics of most patients. No adverse events were observed from performing the index tests.

### Estimates of the relation between presence of patterns and complaints

#### Findings of the primary examiners

Twenty-two patients were referred due to complaints from one and five patients due to complaints from both upper limbs. Among patients referred for other reasons six were currently symptomatic, three had prior symptoms from one or both upper limbs, and five patients had never experienced upper limb symptoms.

Taken together, pain, weakness, and/or numbness/tingling were present in 38 limbs and absent in 44 limbs (Table 3). The primary examiners diagnosed upper limb conditions in 22 of the 38 symptomatic limbs (Table 3).

22 and 5 patients were referred due to complaints from one and both upper limbs, respectively. Among patients referred for reasons other than upper limb complaints, 6 also had complaints from one of the upper limbs. Out of 44 non-symptomatic limbs, previous symptoms were reported by 15. Eight patients had never experienced upper limb symptoms.

#### Findings of the secondary examiners and the relation to symptoms

The secondary examiners agreed on the presence of any of the ten defined patterns in 90 instances (Table 2) in 30 limbs and disagreed in 10 limbs. The patterns could occur in isolation or combined with upper limb conditions diagnosed by the primary examiners (Table 3).

Based on muscle weaknesses, sensory disturbances, and mechanical allodynia, both secondary examiners identified a pattern in accordance with brachial plexopathy in 15 symptomatic limbs for which concurrent presumed pathology was specified by the primary examiners [3] (Table 3): Supraspinatus tendonitis or lesions in eight limbs, frozen shoulder in one limb, one previous clavicular fracture, one tension neck syndrome, cervical syndrome with radiation to three limbs, and lateral epicondylitis in one limb. From the location adjacent to the shoulder in most of these limbs, the patterns identified by the secondary examiners would suggest the presence of a localized nerve affliction which could be associated to the findings of the primary examiners. In 13 limbs neuropathy was indicated by the findings of the secondary examiners but no diagnostic classification made by the primary examiners. The remaining six limbs with disagreement and one limb with agreement on absence by the secondary examiners were diagnosed by the primary examiners as cases of rotator cuff tendonitis, epicondylitis and osteoarthritis.

The prevalence of any pattern being classified as present in each limb was calculated to [30 + (6+4)/2]/82 = 0.43 (Table 2 and 3). With the presence of complaints as reference, the prevalence odds of the combined tests being correct would be 38/44 = 0.86 and the chances in favour 38/(38+44) = 0.46 (Table 2 and 3).

Independently of the diagnoses of the primary examiners (Table 3), the two secondary examiners found one or more pattern present in most limbs with pain, weakness and/or numbness/tingling and excluded their presence in most limbs without these complaints (κ = 0.70 and 0.75, respectively) (Table 4).

There was full consensus between the two secondary examiners with respect to the presence or absence of any defined pattern in 72 out of the 82 limbs (overall inter-rater agreement (42 + 30)/82 = 0.88, κ = 0.75). In these limbs the diagnostic sensitivity and specificity of the combined tests were 0.73 and 0.86, respectively, the positive and negative predictive values 0.93 and 0.90, respectively, and the likelihood ratio 5.2.

For each secondary examiner the corresponding sensitivity was 0.79 and 0.84, respectively, and the specificity 0.90 for both. The positive/negative predictive value and likelihood ratio was 0.83/0.87 and 7.9 for one secondary examiner and 0.88/0.89 and 8.4 for the other (Table 3).

For limbs with full agreement between the two secondary examiners, the post-test odds of 4.3:1 improved the diagnostic confidence from 0.46 to a post-test probability of 0.81. For each examiner, the corresponding post-test odds of 6.8:1 and 7.2:1 improved the post-test probability to 0.87 and 0.89, respectively.

## Discussion

Diagnostic studies usually examine single tests in relation to single reference standards. This study adhered to the clinical practice in the neurological upper limb examination to use multiple tests based on simple semi-quantitative methods and equipment which can be used in any clinical setting.

We have previously documented in a sample of patients in clinical occupational medicine the inter-rater reliability of neurological patterns reflecting the topography and muscular and cutaneous innervation of nerves.

We have now additionally demonstrated that physical findings in terms of identification of patterns in symptomatic limbs differ from findings in healthy limbs. The relation to findings of complaints in 13 limbs out of 30 with agreement in which the primary physical examination did not contribute diagnostically reflects the poor yield of the latter conventional physical approach [11]. Without an assessment in the primary examination protocol of strength, sensibility, and mechanosensitivity it is not possible to identify the neuropathic conditions that according to the secondary examiners appeared to be common. The frequent identification of patterns suggesting neuropathy in limbs in which non-neuropathic conditions were identified by the primary examiners is also noteworthy and suggests the frequent involvement of nervous tissue in various upper limb conditions occurring in an occupational context.

This indication of one aspect of construct validity was achieved in spite of factors that may complicate the physical assessment: Accurate palpation of the peripheral nerves may be difficult due to tissue covering nerves. A potential central nervous system modulation and release of circulating mediators from neurogenous inflammation may cause proximal, distal and even contralateral extension of nerve trunk allodynia. The resulting pain, summation phenomena, hyperalgesia, and expanded receptive fields may all modify and complicate the identification of minor degrees of muscle weakness and sensory abnormalities. An inaccurate and generally less sensitive physical assessment resulting from these difficulties may influence the correlation between findings and symptoms by causing a tendency towards an underestimation of the interrelation.

The identified patterns of weakness associated with appropriately located sensory disturbances and focal mechanical allodynia of nerves were predominantly limited to symptomatic limbs. The arbitrarily defined criteria for the patterns do not allow conclusions with regard to the type and location of pathology responsible for the identified patterns and complaints. To conclude that findings do indeed reflect focal neuropathy with specific locations would demand further studies of validity aspects. However, whether related to pain or not, this study suggests that findings may be explained by peripheral neuropathy with specific locations. In fact they are hard to explain otherwise because the patterns were defined according to the innervation of muscles and skin, and to the course and topography of nerves. Findings are unlikely to represent undiscovered disorders confined to non-nervous tissue, somatization, or malingering. In the absence of neuropathy, patients should be simulating and possess an exact anatomic knowledge which is implausible. Indications of tautology in the concept of myofascial pain and a questioned validity in the phenomenon of trigger points have caused the suggestion that the symptoms with "non-specific" upper limb disorders are rather related to neuropathy [12].

Evaluation of validity depends on the reference selected for comparison. Such selection can be difficult. In comparison to an inaccurate reference, a new test can perform no better than that and may seem inferior even though it approximates the truth more closely. An electrophysiological examination or MRI study aiming to disclose or exclude nerve-afflictions at all the ten studied locations would be comprehensive, time consuming, costly and uncomfortable to patients. But more importantly, to be feasible they should also accurately reflect the target disorders. Measures of nerve conduction velocity are unlikely to recognize minor upper limb neuropathy which is predominantly consisting of partial and mixed lesions with the majority of fibres intact [13,14]. Imaging techniques are also not currently suitable to reflect such pathology and it appears that no laboratory studies can offer a global diagnostic approach to upper limb neuropathy. In contrast, aiming to target specific pathology with specific location(s) any such technique would require a preceding systematic and detailed neurological examination comprising parameters similar to those addressed in this study. In spite of current limitations of diagnostic techniques such as electrophysiological measurements and MRI, they may in selected cases support the clinical diagnoses and decisions relating to various treatment protocols, e.g. surgery.

The insufficient or unknown validity of any potential reference for comparison challenges this research and prevents the assessment of criterion-related validity. Still, the question of construct validity can be addressed through studies of the relation between subjective and physical parameters. There is no single method to determine construct validity, which, however, may be supported by evidence accumulated from several studies. Pain, weakness and/or numbness/tingling are characteristic to upper limb neuropathy and constitute sensitive references for comparison. However, the additional relation of these complaints to non-neural pathology limits specificity.

While isolated symptoms or findings are rarely diagnostic their combination may guide but also bias the diagnostic process. This is a risk with most clinical assessment including the neurological examination. A biased estimation of patterns from the execution in this study of several tests by the same secondary examiner cannot be completely excluded in spite of patterns being defined in accordance with anatomic facts and blinded and independent testing and test interpretation.

The symptomatic patients referred for assessment in occupational medicine did not merely represent a group of chronic pain patients. While some patients presented with long-lasting and major disabling symptoms others have had minor symptoms for a short period of time. The duration of upper limb symptoms ranged from a few months to several years preceding referral. About half of the patients were on sick-leave while the remaining patients were able to continue their work. Most patients with upper limb symptoms were formerly diagnosed with specific disorders such as tennis elbow or shoulder tendonitis. Many had several such diagnoses suggested by various specialists. Others were labelled as non-specific upper limb conditions such as RSI (repetition strain injury). In many patients a neuropathic condition was suspected and electrophysiological studies (mostly of the median nerve in the carpal tunnel) and imaging (especially of the cervical spine) performed. These additional diagnostic studies did not contribute diagnostically. Previous treatment with NSAID, physiotherapy, surgery, etc. had been largely unsuccessful.

The results achieved in this study may be influenced by clinical variables such as the expertise of the examiners. Both learned the techniques of assessment rather recently. After two years of practice one of the examiners supervised the other in assessment of 20 patients before the study. The examination was applied in subjects among whom it is clinically justified to suspect the target disorders. The balanced distribution and wide spectrum of disease in the sample is apparent from the referral pattern and sample-composition (38 symptomatic and 44 asymptomatic limbs with former symptoms in 15, the minor severity in symptomatic patients referred for other reasons than upper limb complaints, and five patients with bilateral symptoms but unilateral dominance). Still, the frequency and severity of upper limb disorders in the sample may influence the external validity.

The agreement on presence in two and disagreement in one out of 29 never-symptomatic limbs may be spurious or explained by a latent neuropathy [15,16]. An indication of the latter might be that at follow-up two years after termination of the study, incident symptoms had occurred in one out of two never symptomatic limbs with unanimously identified patterns. Out of 15 limbs with former symptoms, the examiners disagreed with regard to the presence of patterns in three. Symptoms recurred in two of these at follow-up.

The criteria applied may have been too insensitive to explain upper limb complaints in the four symptomatic limbs in which neither secondary examiner recognized a pattern, or the condition responsible for the symptoms may be untargeted by the physical examination. Symptoms were confined to the innervated territory in five limbs with an isolated distal pattern, but variably located in 25 limbs with patterns suggesting brachial plexopathy. The common identification of patterns suggesting brachial neuropathy is noteworthy, but it should be emphasized that distal neuropathy was additionally suggested in the majority of these limbs. While it would seem to be important from a preventive perspective it cannot be determined from this study whether proximal neuropathy occurred secondary to distal neuropathy or vice versa.

A peripheral nerve-involvement in a proportion of the studied patients is supported by studies of comparable samples such as patients with "non-specific" upper limb disorders. Findings include elevated thresholds to vibration [16], positive upper limb tension tests [9], reduced nerve mobility [17], abnormal nerve tenderness [6], changed axonal flare reaction [18], secondary hyperalgesia [19], allodynic responses to supra-threshold vibratory stimulation [16], chronic compartment syndrome [20], and reduced sympathetic reflexes [21]. The frequency of patterns indicative of brachial plexopathy in the studied sample is supported by the identification in a similar sample of a high proportion of brachial plexus afflictions after the application of a detailed physical examination incorporating the proximal portion of the upper limb nerves [22]. Positive responses to the abduction external rotation test have been reported in a high proportion of symptomatic workers [23] with variable prevalence between occupations [24]. In spite of low specificity [25], it has been demonstrated that provocative tests involving the nerve bundles adjacent to the shoulder joint can predict future upper limb symptoms and signs of neuropathy [26].

## Conclusion

We have studied one aspect of construct validity of a neurological upper limb examination consisting of an assessment of strength in representative muscles, sensory qualities in selected innervation territories and nerve trunk mechanosensitivity at defined locations. Applied to a sample of patients in occupational medicine the examination could identify patterns of neurological findings related to the presence of complaints.

Only an estimated quarter of work-related upper limb disorders can currently be diagnostically classified with a standard physical approach [27]. Taking into account that neurological patterns suggesting peripheral neuropathy were identified in a high proportion of limbs, it is likely that the examination can significantly contribute to the diagnosis in these patients.

Generalization and clinical feasibility of the presented examination, however, demands further studies of validity. First of all, the examination should be tested in samples with different disease prevalence and severity. In addition, further studies of construct validity should deal with the relation of neurological patterns to other features of upper limb neuropathy. Recommendations for treatment or prevention of the conditions identified by the examination would demand a broader evaluation than presented here and possibly MRI and electrophysiological studies may play a role in such assessment. An eventual future demonstration of a beneficial effect of treatment and prevention based on the examination would contribute further to validation.

## Competing interests

The author(s) declare that they have no competing interests.

## Authors' contributions

C-G Hagert has developed the physical diagnostic approach concerning the systematic evaluation of strength in individual muscles and the identification of soreness at potential locations of neuropathy. A I Larsen and J R Jepsen initiated and designed the study. A I Larsen, L H Laursen, and J R Jepsen collected the data. S Kreiner conducted the statistical analyses in cooperation with L H Laursen and J R Jepsen. L H Laursen, C-G Hagert, and J R Jepsen were responsible for the preparation of the manuscript.

## Pre-publication history

The pre-publication history for this paper can be accessed here:



## Figures and Tables

**Table 1:** Rating of physical findings

**Manual testing of isometric strength in individual muscles**
Grading into five levels [28,29]:
• Grade 5 Contraction against powerful resistance, normal power (score = 0)
• Grade 4+ Contraction against gravity and strong resistance (score = 1)
• Grade 4 Contraction against gravity and moderate resistance (score = 2)
• Grade 4- Contraction against gravity and slight resistance (score = 3)
• Grade 3 * Contraction against gravity only (score = 3) *No pareses minor than grade 3 were observed.

**Sensibility to light touch, pain (pinprick), and vibration (tuning fork 256 Hz)**

Grading into three levels:
• Normal (score = 0)
• Mild/any deviation of sensibility (score = 1)
• Marked deviation of sensibility (score = 2)

**Nerve trunk mechanosensitivity assessed by palpation**

Grading into four levels:
• No/normal tenderness (score = 0)
• Mild/any mechanical allodynia (score = 1)
• Medium mechanical allodynia (score = 2)
• Marked mechanical allodynia (score = 3)

**Table 2:** Definition of patterns of neurological findings

**Location of mechanical allodynia**	**Patterns**		**Number of limbs classified in agreement**	
	
	**Reduced strength**	**Sensory deviations**	**Pattern absent**	**Pattern present**
Brachial plexus (Upper trunk level)	Infraspinatus, post. deltoid, biceps	Axillary, musculocutaneous	58	5
Brachial plexus (Cord level)	Post. deltoid, biceps, FCR	Axillary, median, musculocutaneous	48	21
Suprascapular nerve (Suprascapular notch)	Infraspinatus	-	55	4
Axillary nerve (Quadrilateral space)	Posterior deltoid	Axillary	53	20
Musculocutaneous nerve (Coracobrachial muscle)	Biceps	Musculocutaneous	66	4
Radial nerve (Upper arm)	Triceps, ECRB, EPL	Radial	64	13
Posterior interosseous nerve	ECU	-	66	10
Median nerve (Elbow level)	FCR, FPL	Median	54	10
Carpal tunnel	APB	Median	79	1
Ulnar nerve (Elbow level)	FDP V, ADM	Ulnar	74	2

Abbreviations: ECRB = Extensor carpi radialis brevis, ECU = Extensor carpi ulnaris, EPL = Extensor pollicis longus, FPL = Flexor pollicis longus, FCR = Flexor carpi radialis, FDP V = Flexor digitorum profundus to 5^th ^digit, ADM = Abductor digiti minimi, APB = Abductor pollicis brevis

**Table 3:** The relation of integrated findings to symptoms and findings of the primary examiners. Absence or presence of any of the defined patterns of neurological findings identified by the secondary examiners related to upper limb symptoms and to the diagnostic conclusions of the primary examiners

		**Number of limbs in which any pattern was identified by secondary examiners**			
			
**Pain, weakness and/or numbness/ tingling**	**Upper limb diagnoses of the primary examiner**	**Agreement on "absence"**	**Disagreement**	**Agreement on "presence"**	**Total number of limbs**
	
**Absent**	**Absent**	**38**	4	2	44
	
**Present**	**Present**	1	6	**15**	22
	**Absent**	3	-	**13**	16
	
**Total number of limbs**		42	10	30	82

**Table 4:** The relation of integrated findings to symptoms. Absence or presence of any of the defined patterns of neurological findings identified by the secondary examiners related to prior and present upper limb symptoms and to the background for referral

		**Number of limbs in which any pattern was identified by secondary examiners**			
			
**Pain, weakness, and/or numbness/tingling**		**Agreement on "absence"**	**Dis-agreement**	**Agreement on "presence"**	**Total number of limbs**
**Absent (44 limbs)**	**Never**	**26**	1	2	29
	**Former**	**12**	3	-	15

**Present (38 limbs)**	**Referred for other reasons**	1	1	**4**	6
	**Referred due to symptoms**	3	5	**24**	32

**Total number of limbs**		42	10	30	82
